# The Integral Role of Diets Including Natural Products to Manage Rheumatoid Arthritis: A Narrative Review

**DOI:** 10.3390/cimb45070341

**Published:** 2023-06-28

**Authors:** Ayse Gunes-Bayir, Beyza Mendes, Agnes Dadak

**Affiliations:** 1 Department of Nutrition and Dietetics, Faculty of Health Sciences, Bezmialem Vakif University, 34065 Istanbul, Turkey; 2 Institute of Pharmacology and Toxicology, Clinical Pharmacology, University of Veterinary Medicine Vienna, 1210 Vienna, Austria

**Keywords:** Rheumatoid Arthritis, nutritional management, natural products, probiotics, diets

## Abstract

Genetic and environmental factors including lifestyle are thought to play a key role in the pathophysiology of rheumatoid arthritis (RA). There is evidence that diet can enhance the inflammatory response in genetically predisposed individuals. On the other hand, certain types of diets can alleviate RA symptoms due to their anti-inflammatory and antioxidant activities. Also, natural compounds with potential effectiveness in RA management belong to different chemical classes such as flavonoids, polyphenols, carotenoids, and alkaloids with their antioxidant characteristics as well as probiotics. The nutritional approaches to prevent or extenuate the disease progress were examined in this narrative review which was conducted using the PubMed, ScienceDirect and Google Scholar databases and conforms to the Scale for the Assessment of Narrative Review Articles (SANRA) guidelines. Mediterranean and vegan diets equally have been shown to exhibit positive effects on RA as the consumption of dietary fiber, antioxidants and anti-inflammatory compounds from fruits, vegetables, grains, nuts, and seeds are high. Whereas Mediterranean diet additionally includes beneficial nutrients of animal origin such as omega-3 polyunsaturated fatty acids from fish and seafood, patients on vegan diet need to be monitored closely for intake of all critical nutrients. Certain calorie restrictions and intermittent fasting diets have been shown to benefit RA patients although there is an obvious need for further studies to establish solid evidence-based recommendations and guidelines. The research data available strongly suggest that dietary approaches with anti-inflammatory properties may help delay the onset of RA and/or improve symptoms and thus nutrition should be routinely addressed to facilitate management of the disease.

## 1. Introduction

Among the non-communicable diseases, the most common chronic diseases of the musculoskeletal system are rheumatic diseases [1]. Rheumatoid arthritis (RA), which is one of the rheumatic diseases, is an autoimmune, progressive, systemic inflammatory disease characterized by joint destruction, affecting approximately 1% of the world population [2]. The prevalence of RA has a higher prevalence in industrialized countries [3].

Genetic factors depending on demographics and socioeconomics as well as exposure to environmental risk factors promote the development of RA. In 2019, the incidence of RA in Ireland was 38.6 per 100,000 people, the highest incidence in Europe [4]. In the United States, 24% of all adults, equivalent to a population of 58.5 million, suffer from arthritis [5]. Knees, hands, and wrists are commonly affected [6]. Symptoms can range from mild to severe swelling, pain, stiffness, and decreased range of motion [7]. In severe cases, chronic pain and permanent joint damage can result in deterioration in quality. Chronic pain, which has a multidimensional structure, comes first among the complaints of patients with rheumatism [8]. Therefore, The American College of Rheumatology Pain Management Task Force has recognized that pain management is a critical aspect for patients with rheumatic diseases [9]. Pain management for RA is applied according to the patient, taking into account age, comorbidity, specific rheumatic process, related pain mechanisms, and the patient’s personal beliefs. Patients suffering from this disease may experience disability at an early age and problems due to the side effects of the drugs used [10].

Although the etiology of the disease is not exactly known yet, genetic factors are responsible for some of the risks of the disease [11]. In general, the underlying cause of the disease is considered to be multifactorial since it is a combination of genetic, hormonal, and environmental factors [12]. Shared epitope (SE) alleles are thought to be responsible for approximately 40% of the genetic risk. SE alleles are associated not only with RA susceptibility but as well with the severity of the disease, and they can help to identify different genetic profiles in subsets of RA patients [13]. In women, hormonal factors such as estrogen bioavailability contribute to the development of RA. It has been shown that early menopausal age, periods of postmenopausal and postpartum, and the use of anti-estrogen agents are associated with the onset of RA. The impact of systemic hormonal treatments including contraceptives on RA development remains unclear [14].

Lifestyle risk factors including smoking, passive exposure to cigarette smoke, excessive coffee intake, inadequate nutrition, low in antioxidants, and high in red meat consumption can increase the risk of developing RA [15]. There is a need to find strategies for optimizing potential nutritional and pharmacological synergistic impacts in order to facilitate the management of RA. Therefore, it was the aim of this narrative review to assess the available evidence of nutritional approaches with anti-inflammatory properties to prevent or mitigate the progression of rheumatoid arthritis and highlight the benefits and risks of particular diets.

## 2. Methods

This narrative review conforms to the Scale for the Assessment of Narrative Review Articles (SANRA) guidelines [16]. The PubMed/Medline and ScienceDirect databases were searched for articles containing the main keywords (“rheumatoid arthritis” OR “RA patients” OR “Diets in RA patients” OR “Diets in rheumatoid arthritis”) AND (“Mediterranean diet in RA patients” OR “Elimination diet in RA patients” OR “Vegan Vegetarian diet in RA” OR “Calorie restriction in RA” OR “Fasting in RA”) AND (“Natural products in rheumatoid arthritis”) published in English published studies, between January 2003 and June 2023. Secondary search engines (e.g., Google Scholar) were also utilized and 1,020,000 scientific articles were found. Based on extensive searches of scientific sources, the relevant published reports with a strong focus on more recently published studies which contain the main keywords were included in this narrative review. Pre-clinical, clinical, observational or case-control, prospective cohort studies, as well as reviews and meta-analyses were investigated. No exclusion criteria based on the participant’s gender or age were applied.

## 3. Pathophysiology of RA

Along with the individual’s environmental factors and genetic predisposition, lifestyle factors such as smoking support RA development [17]. Amongst other aspects of the complex pathophysiology of RA which is not described here in detail, there is evidence of gene-environment or gene-lifestyle interactions. Studies report increased incidence of RA in cigarette smokers bearing the human leukocyte antigen (HLA)-DRB1 shared epitope [18]. It is suggested that cigarette smoking modulates the immunogenicity of citrulline and related peptides in individuals with specific HLA alleles. Interaction of smoking and arthritis in the context of genes and environment but suggesting that inhaled non-nicotinic components of cigarette smoke are more important than nicotine itself in the etiology of chronic inflammatory diseases [19]. Criteria for classification of moderate and heavy alcohol consumption were based on dietary guidelines for Americans [20]. An association between high and moderate alcohol consumption and increased markers of inflammation was found [21]. On the other hand, negative effects of alcohol intake on the immune system vary widely. Patients with axial spondylarthritis (axSpA) performed study compared the alcohol drinker group and the non-drinker group [22]. It was found that an association between alcohol consumption and spinal structural progression in axSpA patients.

Almost two decades ago it has been shown that citrullination is an inflammation dependent process [23]. RA patients generate anti-citrullinated protein antibodies (ACPA) which can be detected with high specificity in the sera of most RA patients. The anti-citrullinated humoral response is suggested to be strongly linked to inflammation and ultimately RA development [24]. It is unknown whether only a few citrullinated proteins drive the complete ACPA response in RA or whether unique ACPAs exist for each protein [25]. It was just recently suggested that some oral and gut microbial species (e.g., *Porphy-romonas gingivalis*, *Prevotella copri*) may also contribute to RA pathogenesis [26]. Intestinal microbiota dysbiosis occurs in the preclinical stage of RA and is closely associated with the onset of arthritis [27]. Immune dysregulation and changes in metabolic pathways play a central role regarding RA risk and progression.

*Porphyromonas gingivalis* is a natural component of the oral flora and associated with the development of periodontitis in individuals. Antibodies to *P. gingivalis* have been found to be associated with anticitrullinated protein antibodies in patients with RA suggesting that this bacterium may play a role in RA [28]. The ability of *P. gingivalis* for citrullination through post-translational modification of arginine residues emerges using peptidylarginine deiminases. Many autoimmune diseases including RA produce autoantibodies that recognize post-translationally modified peptides [29]. In an in vitro study, *P. gingivalis* increased the apoptosis of chondrocytes and interfered with the cell cycle, thereby impairing cartilage integrity and transformation [30]. Additionally, it has been demonstrated in in vitro as well as in vivo studies that RA may spread from joint to joint in association with circulatory migration of synovial fibroblasts [31,32]. On the other hand, during dysbiosis, these bacteria can mediate the citrullination of either human or bacteria proteins to trigger an immune response that leads to the generation of autoantibodies [26]. Some pathogenic bacteria such as *Prevotella copri* are suggested to cause gut dysbiosis and stimulate the inflammatory components cascade in the gut tissues. *P. copri* exhibits pathogenic properties associated with RA patients. Its effects are not restricted to altering the intestinal microbiota and permeability but extended to induce pro-inflammatory effects [33]. A study showing the contribution to the development of joint inflammation demonstrated that when *P. copri* becomes predominant in the intestine, autoreactive T cells be-come highly reactive to arthritis-related antigens via activation of innate immunity [33]. These T cells can then induce joint inflammation. In this context it is important to RA patients to maintain a functional microbiome also by means of dietary approaches which may help to delay the onset of RA and/or improve symptoms.

## 4. Pharmacotherapeutic Management of RA

Pharmacotherapy of RA includes molecules that provide symptomatic relief in RA patients and slow the progression of the disease [34]. Although the pharmacotherapeutic management of RA is complex, it relies on a range of medical evaluations, evaluation of the safety and efficacy profiles of molecules, and straightforward interpretation of the results of evidence-based clinical and paraclinical studies, according to the recommendations of international therapeutic guidelines.

Treatment of RA generally consists of anti-inflammatory medication [35] to stop the inflammatory response [36] by non-steroidal anti-inflammatory drugs (NSAIDs) and disease-modifying anti-rheumatic drugs (DMARDs). A better understanding of the pathophysiology and pathogenesis of the disease has led to the use of advanced drug treatment strategies including antibody therapies which improve the quality of life of patients affected [37]. A treat-to-target approach with synthetic and biologic disease modifying anti-rheumatic drugs yields superior outcomes to standard care in RA but full implementation remains uncommon in the clinic [38]. The expected aim of these treatments is disease remission or reduction of severity in the early stages of the disease. Nevertheless, clinical trials have shown that a significant proportion of RA patients event inefficacy treatment and/or side effects by multifarious anti-RA drugs [39]. Especially hepatorenal toxicities, cardiovascular diseases and other side effects limit the use of these drugs in clinical practice.

## 5. Natural Products and RA

Therapeutic and preventive impacts of some plants and their compounds in RA management were reported [40]. Chemical classes of natural compounds for RA management include flavonoids (luteolin), stilbenoidpolyphenol (resveratrol), anthraquinone glycoside (emodin), caffeic acid ester (rosmarinic acid), phenolic xanthonoid (mangiferin), carotenoids (β-cryptoxanthin), and alkaloids (piperlongumine), etc.

A recent meta-analysis [41] turned out that increased caffeine consumption was not associated with an increased risk of RA, but coffee consumption per se did show positive relation to the risk of RA. The authors suggested that along with caffeine, other chemicals used in the cultivation and processing of the coffee may also be managing RA risk. This hypothesis is corroborated by the findings that decaffeinated coffee also increased the risk of RA. It has been asserted that caffeine and coffee may have positive and negative interactions on RA symptoms and comorbidities but might also change the kinetics of some drugs used in the treatment of RA [42]. No significant association was found between tea consumption and RA risk. An earlier meta-analysis reported that based on the studies used it could not be found that tea consumption significantly decreased RA incidence, despite an efficacy of tea on RA risk is biologically reasonable [43]. On the contrary, in a recent study tea consumption has been demonstrated to be inversely proportional to the risk of RA [44]. This protective effect of tea consumption may be due to the antioxidant properties of tea.

Green tea is known to be rich in polyphenols with epigallocatechin-3-gallate (EGCG) being the major component [45]. EGCG reveals anti-inflammatory and anti-arthritic activities due inhibiting enzymes and signaling which play substantial roles in inflammation and joint destruction in arthritis. The regulative effect of EGCG on RA can occur through the expression of cytokines, chemokines, matrix metalloproteinases (MMPs), aggrecanase, reactive oxygen species (ROS), nitric oxide (NO), cyclooxygenase-2 (COX-2), and prostaglandin E2 (PGE2). Although the exact etiology of RA remains unknown, several studies have confirmed the role of ROS in the pathophysiology of the disease causing inflammatory responses [46]. ROS are naturally produced during aerobic metabolism, and the cells are protected against ROS by the antioxidant defense system. Excessive production of ROS causes oxidative stress, metabolic dysfunction and damage to cells. Therefore, using antioxidant supplements such as EGCG or increase green tea consumption in the regular diet may help reduce the symptoms and improve the quality of life in RA patients based on the free radicals produced from oxygen metabolism destroying the antioxidant system. A rat study comparing green tea with black tea found that green tea could reduce rheumatic manifestations with an anti-arthritic activity comparable with that of indomethacin [47]. In this experimental study, rats treated with green tea has shown highly reduced clinical arthritis in comparison to black tea-treated and untreated rats. When the serum of rats given green tea was analyzed, pro-inflammatory cytokine levels such as tumor necrosis factor alpha (TNF-α) and interleukin 1 beta (IL-1β) were found to be significantly reduced. In other studies, it has been reported that green tea consumption is a preventive factor for RA, and long-term consumption of green tea can reduce the risk of RA onset by 35% [48].

To alleviate rheumatic pain some patients, tend to seek additional help from complementary medicine (CAM). In a national survey of rheumatologists practicing in the United States, 54% reported acupuncture beneficial for pain management [49]. However, there are currently no gold rules and standards for CAM applications in the treatment of RA [7]. Almost 50% of patients never inform their physicians about their CAM treatments [50]. Survey based the most frequent causes of not telling is that patients were not asked about the use of CAM by physicians or forgot to tell the doctor and some patients feared disapproval. In a study, it was determined that approximately 33% of adults and 12% of children benefited from CAM in the treatment of chronic pain in the USA [51]. In the UK, CAM therapies in arthritis patients have increased from 38% to 60% [7,52]. Herbal products and phytotherapy are widely used in CAM. Nature has been a source of medicinal agents for more than thousands of years and herbal therapy predominates in traditional systems of medicine as well as in alternative medicine practiced in various cultures in the world [53,54]. Studies suggest that polyphenols, phenolic acids, alkaloids, and triterpenes can alleviate the inflammation of diseases such as RA due to their anti-inflammatory properties [53]. For example, flavonoids as ubiquitously occurring polyphenolic compounds occur in many plant foods such as vegetables, fruits, tea, and cocoa [51]. In this regard it is not surprising that in addition to drug treatment, the prognosis of this disease can be alleviated with various nutritional approaches and physical therapy [35,36]. Herb-herb combinations also known as polyherbal formulations have been used in Asia traditional medicine, which provide treatment of diseases in a holistic approach, but scientifically their therapeutic benefits are lacking, and it has not been explored [54].

## 6. Nanomedical Management of RA

Nanotechnology comprises science and technology based a combination of several fundamental sciences (e.g., chemistry, biology, physics, and mathematics) and technology of nanometer-scale objects [55]. Significant increase in nanotechnology research for the treatments of RA, because of the possible improvements were observed over the conventional systemic treatments [56]. Various types of nanomaterials have been investigated (i.e., liposomes, polymeric micelles, metallic nanoparticles, solid lipid nanoparticles, magnetic nanoparticles etc.) in numerous in vitro and in vivo studies. Especially, in vivo studies were performed in different routes of application on rats and mices. There is still more research required on the final clinical translations of nanotherapies against RA and clinical implementation.

## 7. Diets and RA

In general, many of the foods frequently consumed in the western world bear the risk of acting as a co-contributor in the pathogenesis of RA. In this regard it has been suggested that permanent consumption of sugar-sweetened carbonated beverages may be connected with a rise risk of seropositive RA. Further epidemiological reports are needed to confirm these findings and to reveal the potential biological mechanisms [57]. According to the results of a study conducted in the United States, regular consumption of beverages high in free fructose has been associated with arthritis in adults. It has been observed that there is a link between regular high free fructose consumption (at least 5 times a week) and the prevalence of arthritis [58].

According to the international definition, probiotics are live microorganisms that provide health benefits to the host when administered in adequate amounts [59]. Probiotics are organisms that greatly affect the development and performance of the immune system that provides intestinal homeostasis. It shows a therapeutic effect of probiotics in the right amount and depending on the microorganism strain in individuals [60]. In RA patients, probiotic supplementation has been shown to give patients an overall advantage in a shorter time. Some studies have suggested that the use of probiotics may be beneficial to reduce the risk of developing arthritis or reduce the arthritic process [61,62]. Gut microbiota was also found to positively influence the balance between pro- and anti-inflammatory immune responses during collagen-mediated induction [62]. Particularly, there are reports for the utilization of *Lactobacillus casei* or *Lactobacillus acidophilus* probiotics in the treatment of RA [60].

**Figure 1:**
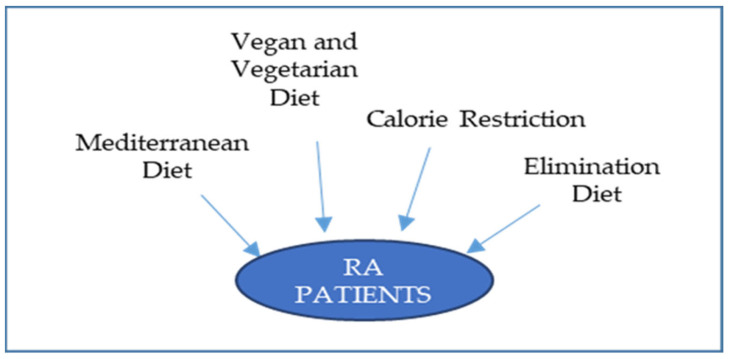
Diet types to manage RA symptoms in RA patients. RA: Rheumatoid arthritis.

Diet is one of the foremost easy changeable environmental factors playing an important role in RA [63]. Different types of diets to manage symptoms of RA patients are demonstrated in Figure 1. Experimental studies show that calorie restriction or intermittent fasting are beneficial in slowing or preventing chronic, degenerative, and inflammatory diseases.

Diet changes or restrictions have long been recognized as appropriate approaches and practiced clinically to supplement or complement pharmacological treatment strategies [1,64]. Table 1 shows the recommended foods to be consumed in different diet types to manage RA symptoms. Dietary changes can be an easy and well-balanced approaches in the management of the disease [6].

In addition to standard drug therapy, fasting as a complementary treatment, Mediterranean diet, Cretan Mediterranean diet, vegetarian diet as anti-inflammatory diets and the use of various specific foodstuffs on RA have been examined in various studies [65]. Fasting followed by the Mediterranean diet, the Cretan Mediterranean diet as well as anti-inflammatory diets have been shown to have a beneficial effect on the treatment of RA. It was seen that diet may help in the management of RA as a supplementary part to standard drug therapy. A systematic review/meta-analysis was investigated the effect of anti-inflammatory diets including Mediterranean diet, vegan and vegetarian diets on pain in RA [66]. It was reported that such anti-inflammatory diets resulted in significantly lower pain than ordinary diets. Table 2 demonstrates a summary of the reviewed papers for the evaluation of the effectiveness of these diets as a nutritional intervention in the management of RA symptoms. There is limited evidence of an association between the Mediterranean diet and RA symptoms, with the Mediterranean diet alleviating RA symptoms.

### 7.1. Mediterranean Diet

Clinical studies have proven that the beneficial effects of the Mediterranean diet are especially effective in fighting obesity, cancer, cardiovascular and metabolic diseases, based on its anti-inflammatory and antioxidant properties [73]. The relationship between inflammation and atherosclerotic disease is verified [74]. On the other hand, recent evidence suggests that inflammation may also play a role in the development of non-ischemic heart disease in RA patients as well as an increased risk of mortality from cardiovascular disease.

Mediterranean diet contains high amounts of cold pressed extra virgin olive oil, fresh and cooked vegetables including legumes, dried and fresh fruit, nuts, unrefined grain products, milk and dairy products, fish and seafood, poultry, small quantities of red meat and sweets, and moderate salt and red wine consumption [75]. In the western-type diet however, people often consume foods high in saturated fat because these are fast and easy to prepare. The common western diet is qualified by high consumption of saturated fat, nitrite, nitrate, and iron, along with excessive consumption of red meat and other products able to increase inflammation [76]. Red meat meals contain nitrites and nitrates, which are often carcinogenic and pro-inflammatory.

In a randomized controlled trial, a proportion of 44 RA patients received the Mediterranean diet and residue of patients applied a standard healthy diet for 12 weeks [77]. Mediterranean diet for this RA patients was rich in legumes, unsalted unroasted nuts, fermented dairy products, fish and poultry, but contained small amounts of red meat. RA patients in the healthy diet group were recommended to consume 5–7 servings of fruit and vegetables a day, and to consume foods rich in fat, sugar and salt once or twice a week. As a result of this study, it was seen that the Mediterranean diet improved the physical function and quality of life of the patients with RA. Additionally, sodium intake due to consumption of high amounts of salt is also associated with RA [78].

Western diet patterns usually lead to a disproportion in the ratio of omega-6 to omega-3 polyunsaturated fatty acids (PUFA) in favor of omega-6 [79]. A higher inflammation level is caused by higher intake of omega-6 PUFA which is resulted in consumption of a high saturated fat or foods with a high glycemic load and in consumption of lower dietary fiber [80]. In contrast to this, intake of long-chain omega-3 PUFA, obtained from fish and seafood, as frequently consumed in Mediterranean diet, is related to a reduced risk of RA presumably due to their anti-inflammatory features [81]. Omega-3 PUFA are not only found in fatty fish and fish oil supplements but also nuts e.g., walnuts and seeds e.g., flaxseed, soybeans, eggs, avocados and e.g., cauliflower [76].

Recently, a systematic review showed that anti-inflammatory-based dietary interventions can be an effective avenue for adults with RA seeking complementary therapies, potentially leading to improvements in certain parameters [82]. However, it indicates that the anti-inflammatory benefits of a diet in combination with omega-3 PUFAs may be superior to diet alone, warranting further research in this area.

The Mediterranean diet is not only recommended on account of the immune modulatory properties of mono- and polyunsaturated fatty acids consumed but also because of the variety of antioxidants, vitamins, and polyphenols in the diverse vegetables and fruits [75]. Especially olives and olive oil are rich in polyphenols and so is red wine. Resveratrol, the main polyphenol in red wine, has well-established anti-inflammatory effects. It has just recently been suggested that resveratrol has healer effects on RA through signal transducer and activator of transcription/Hypoxia inducible factor-1/Vascular endothelial growth factor (STAT3/HIF-1/VEGF) signaling pathways [83].

The Mediterranean diet is consisting of various biologically active foods. Consuming fruits and vegetables such as sweet potato, carrot, capsicum, pumpkin, avocado, watermelon, oranges and pomegranate rich in carotenoids has been shown to be beneficial in the treatment of RA due to the antioxidant and anti-inflammatory properties of these functional pigments [84,85]. Carotenoids such as β-cryptoxanthin, lycopene, β-carotene, α-carotene, zeaxanthin, and lutein reveal their antioxidant properties due to inhibiting ROS and their anti-inflammatory properties due to lowering C-reative protein levels, thus exerting a protective effect in the management of RA [85,86]. Natural products including food pigments such betalains in red beet also have shown to possess the ability to significantly decrease pro-inflammatory cytokines and subsequently induce positive effects in RA patients [87]. Rosmarinic acid is commonly found in spices often used in the Mediterranean diet, such as rosemary and basil [88]. It has been shown that rosmarinic acid have an apoptotic and anti-inflammatory effect against T cells in RA cases [89].

A few research has proposed that higher intake of fish, cooked vegetables, and olive oil in the daily diet is associated with a reduced risk of developing RA. Especially, dietary fiber content is remarkably high in this type of diet [1]. Nutrition connects the intestinal microbiota and immune system responses with inflammatory diseases by various molecular mechanisms [67]. One of these describes that intake of high dietary fiber encourages modifications of intestinal microbiota with decreasing Firmicutes and increasing Bacteroidetes, which produce elevated levels of short-chain fatty acids (SCFA). SCFA supports intestinal homeostasis by promoting mucus secretion by intestinal epithelial cells [68,90]. The mucus barrier makes an important contribution to both intestinal homeostasis and immune tolerance. In this regard metabolites such as SCFA has a critical role in the prevention of inflammatory disease and show a link with the immune system, which may clarify the relationship between inflammatory diseases with diet and intestinal microbiota. SCFA production increases as more dietary fiber is ingested. However, the precise characteristics of a healthy microbiota have not been completely explained [67]. Because the Mediterranean diet is rich in fiber, it may have positive effects on the changes in microbiota composition of RA patients [69]. In the same way, a vegan or vegetarian diet may be beneficial for RA patients [6]. The Mediterranean diet is not only related with health benefits, moreover it is delicious and offers variety, which can be adopting it as an eating pattern and lifestyle helps it fit longer lifestyle [91]. However, studies on Mediterranean diet and RA are limited and more studies are needed.

### 7.2. Calorie Restriction

Calorie restriction and intermittent fasting have shown potential benefits in patients with RA although studies are rare [36,92,93]. Calorie restriction is characterized by a consistent reduction on average daily caloric intake of approximately 500–800 kcal without causing malnutrition, whereas during intermittent fasting patients’ cycle through periods of drastically cutting food intake or drastically limiting calories and periods of healthy eating. Types of intermittent fasting include 16/8 fasting (healthy eating limited to one 8-h window per day), 5:2 fasting (healthy eating for 5 days per week, and for 2 days limiting calorie intake to 500), alternate day fasting (fasting every other day) or one meal a day diet (fasting for 23 h and eating daily during a 1-h window) [92]. It has been known for years that fasting and calorie restriction accelerate autophagy and cellular clearance [93]. Autophagy, classified as macro, micro, and chaperone-mediated, is a process of cellular homeostasis and lysosomal biodegradation that the body uses against internal and external stressors [94].

There are many studies that intermittent fasting is associated with slowing or preventing chronic inflammatory diseases [95]. Intermittent fasting can increase immune functions by downregulating pro-inflammatory cytokine expression [36]. In a study, it was found that 12-h fasting in mice increased beta-hydroxybutyric acid levels, thereby regulating forkhead box O1 (FOXO1) transcription factor and heme oxygenase 1 (HO1), an antioxidative enzyme [96]. Beta-hydroxybutyric acid also has been shown to prevent cell damage by decreasing inflammatory responses and apoptotic cell death due to down-regulation of NFkB and NLRP3 inflammasome. NFkB plays a critical role in inflammatory processes as a major transcription factor. In studies, a modified intermittent fasting diet for 7–10 days followed by a diet containing plant foods has shown positive effects such as reduction in pain and increased function in RA patients [97]. Generally, medical fasting has been found to have a clinically beneficial effect in arthritis patients followed for at least 3 months. Therefore, available evidence suggests that a vegetarian diet after fasting is beneficial in the treatment. Recently, a study revealed that prolonged fasting subsequently a plant-based diet may a new dietary intervention for patients with RA. In this exploratory study, fasting relieved symptoms of RA in 7 days and this benefit was sustained for up to 6 months depending on a plant-based diet [36]. Further studies are needed to validate onset and sustainability of fasting-induced clinically beneficial effects in RA patients.

### 7.3. Vegan and Vegetarian Diets

A vegan diet is a type of plant-based diet which eliminates any animal-origin food. In contrast, some types of vegetarian diets remove meat and fish but the consumption of milk, dairy products, and eggs were included. Since milk and fish are refrained in vegan and some vegetarian types of diet, it is important to supplement vitamin D in order to ingest adequate amounts of this vitamin which not only is important to help absorb calcium and phosphorus from food but also plays a role in the immune system. Its deficiency can interfere with antibacterial and anti-inflammatory responses of immune system. As such, a lack of vitamin D may play a role in the development of RA [97] and counteract the benefits of vegetarian diets including vegan. Based on the results of a study conducted on mice with vitamin D receptor (VDR) deficiency, it was found that inflammation increased, leading to a pro-inflammatory monocyte phenotype associated with cartilage damage [98].

In different studies, it has been revealed that the risk of autoimmune diseases is lower in the vegan diet because there are no animal products consumed. Vegetarian diets contain less arachidonic acid than diets with high or moderate intake of meat, whereas vegan diets contain virtually no arachidonic acid [66]. Arachidonic acid is an essential fatty acid derived from endogenous and exogenous sources. It contributes to the production of eicosanoids and several of them have pro-inflammatory efficacy. Since it is converted into various mediators, such as prostaglandin E2 (PGE2), which engages in the development of RA, the consumption of exogenous sources (nutrients of animal origin) should be minimized. It was confirmed by population studies that a high consumption of foods of animal origin in the Western diet is associated with the event of RA [99]. In this case, it suggests that people with a vegan diet may have a low risk of RA [100].

As mentioned earlier, fruits and vegetables are high in polyphenols, organic compounds that have been shown to alleviate the inflammation process in diseases such as RA [53]. It has been reported that oral application of 1 g of resveratrol co-administered to RA patients with their conventional drug improved the disease induced symptoms by reducing clinical markers (i.e., the 28-joint count for swelling and tenderness) as well as serum levels of certain biochemical markers (i.e., C-reactive protein, TNF-alpha, and IL-6) [101].

Another polyphenolic compound in legumes is genistein which also has anti-oxidative activity. It has been shown to help to reduce reactive oxygen species in RA patients [102]. In contrast, increased consumption of nutrients of animal origin and decreased intake of dietary fiber may increase the risk of developing autoimmune disease [6]. Plant-based diets can reduce the inflammatory response by lowering C-reactive protein (CRP) levels, alleviating joint inflammation and pain and are also low-fat and fiber-rich diets, which can develop the composition and the variety of intestinal bacteria in patients with RA [103].

Increased release of pro-inflammatory cytokines such as interleukins (IL-1, IL-6, and TNF-α) can lead to chronic inflammation [104]. In this regard gut microbiota plays a significant role in the physiological and immunological homeostasis. There are approximately 10–100 trillion microorganisms in the human intestine [71] representing a dynamic microbial ecosystem, with bacteria in the gut varying between 500–1000 different species [72]. Disruption of gut microbiota homeostasis may be associated with various inflammatory diseases, including RA [97]. As mentioned earlier, adequate dietary fiber intake may have a positive effect on the microbiota. Fibrous foods in the daily diet are reduced to SCFA by commensal bacteria. Butyrate, a SCFA, decreases intestinal permeability and bacterial translocation, increasing tight junction protein expression which limits local and systemic inflammation [1,90].

It is known that patients with RA are at higher risk for cardiovascular disease and have higher plasma oxidized low-density lipoprotein (LDL) levels. There is a positive correlation between the development of RA and total cholesterol levels in females but not in males. The effect of total cholesterol on improvement of subsequent disease in women has been shown to be significant for seropositive as well as seronegative RA [12]. A study revealed that vegetarians had lower plasma lipids compared to omnivore counterparts, with the lowest levels reported among vegans [70]. In that study, plasma total cholesterol and LDL cholesterol were 32% and 44% lower among vegans than among omnivores. A higher consumption of nutrients rich in fiber and antioxidants is associated with lower blood cholesterol concentrations [105].

In contrast, increased animal products in the diet and decreased dietary fiber may increase the risk of developing autoimmune disease [6]. Research data show that a low-fat vegan diet recovers some RA symptoms, such as pain rating, joint tenderness and swelling. Moreover, a randomized clinical trial showed that a gluten-free vegan diet reduced the level of immunoglobulin G in patients with RA, thereby improving the signs and symptoms of RA [106]. In studies conducted with RA patients on a vegan or vegetarian diet, a remarkable development in disease symptoms was observed [35]. Nevertheless, it needs to be stated that nutrients deficiencies may arise depending on the restriction of all foods of animal-origin. Hence it seems important to supervise RA patients on vegan diet and provide the correct intake of critical nutrients (e.g., protein, vitamin D, vitamin B12, omega-3 polyunsaturated fatty acids) to avoid impairment of physiological functions.

### 7.4. Elimination Diet

Dietary intake of some individual allergenic foods in RA increases pro-inflammatory processes [35]. RA symptoms may improve due to elimination of allergenic foods and subsequent decrease in inflammation [107]. Foods that are usually triggering the disease should be eliminated from the diet, such as dairy products, meat, corn, wheat, oats and rye, eggs, citrus fruits, potatoes, tomatoes, and coffee, and only non-trigger foods should be consumed [35].

Reducing the intake of food antigens leads to reduction in immunoreactivity. When these foods were eliminated in the diet there appeared to be improvements in the number of tender joints but also in inflammatory biomarkers during the elimination phase, such as in erythrocyte sedimentation rate, CRP, TNF, and IL-1 beta [108]. Recommending a healthy diet based on anti-inflammatory fruits, vegetables, whole grains, and seeds may help to fight inflammation and joint pain [109]. Studies revealed that the removal of allergenic foods from the diet resulted in the recovery of RA patients [106]. However, when individuals return to their normal diet, the disease returns to its former state [110]. A study on a small group of RA patients suggests that in some people the disease may originate from the gut and that RA may emerge as a reaction to some food antigens [111]. The kind of food allergens that trigger RA symptoms show individual differences in patients. There is a lack of data from controlled studies. Therefore, there is insufficient evidence to generalize which trigger foods should be excluded from the patients’ diet [112].

## 8. Limitations

This study was conducted under narrative review approach. Search method and inclusion criteria for this narrative review rely on authors’ experiences and thus may involve subjective selection bias.

## 9. Conclusions and Future Perspectives

Although the etiology of rheumatoid arthritis is not clear yet, research has shown that the pathogenesis of the disease includes genetic, hormonal, and environmental factors. One of the foremost environmental factors is nutrition which has a very important role in preventing the development of RA or extenuating the symptoms in patients suffering from this autoimmune disease. On the other hand, an inadequate diet rich in e.g., saturated fat and sugar, and low in antioxidants can enhance the inflammatory response in genetically predisposed individuals. The Mediterranean type of diet has positive effects on RA as the consumption of red meat and sugar is low and intake of omega-3 PUFAs, polyphenols, vitamins, and dietary fiber is high. This type of diet offers a wide variety, which can facilitate adopting it as an eating model and lifestyle for patients suffering from rheumatoid arthritis. Vegetarian and vegan diets equally have positive effects on RA as the consumption of vegetables, fruits, dietary fiber, and antioxidants are high. In all three types of diet, homeostasis of the intestinal microbiota has been shown to be maintained, and inflammation subsequently can be diminished. Unlike some types of vegetarian diet, vegan diet refrains from all animal products including dairy products and eggs. Therefore, meeting daily protein as well as other critical nutrient requirements needs to be closely monitored in RA patients choosing this type of diet. Elimination of allergenic foods from the individual diet of patients may also help to ameliorate RA symptoms. Calorie restriction and intermittent fasting have been found to have a clinically beneficial effect in arthritis patients although studies are rare. There is an obvious need for further studies to establish solid evidence-based recommendations and nutrition guidelines for patients suffering from RA. Till then it is recommended not to neglect nutrition and the integral role of diet in RA since the research data available strongly suggest that dietary approaches with anti-inflammatory properties may help delay the onset of rheumatoid arthritis and/or improve symptoms and thus nutrition should be routinely addressed in order to facilitate management of the disease.

Diets presented in this narrative review and the evidence-based complementary and alternative medicine (CAM) results may further benefit RA patients. In addition, considering that probiotics are generally accepted as safe (GRAS), the use of probiotics together with the diet suggests that the foods that may be most beneficial in slowing the progression of arthritis. There is a lack of research on dosages of both natural products and probiotic strains. Therefore, it is necessary for researchers on RA to conduct studies on these subjects, which are a part of CAM.

## Figures and Tables

**Table 1:** Food consumption in Mediterranean, vegetarian, vegan and elimination diets.

Mediterranean Diet	Vegetarian Diet	Vegan Diet	Elimination Diet
various fresh and cooked vegetables including legumes	various fresh and cooked vegetables including legumes	various fresh and cooked vegetables including legumes	foods that are usually triggering the disease should be eliminated from the diet; NO coffee
various fruits	various fruits	various fruits	non-trigger foods should be consumed
mainly cold pressed extra virgin olive oil	various plant-based oils	various plant-based oils	various plant-based oils
fish and seafood	YES/NO fish and seafood	NO fish and seafood	YES/NO fish and seafood
dairy products	YES/NO dairy products	NO dairy products	NO dairy products
eggs	YES/NO eggs	NO eggs	NO eggs
poultry	NO poultry	NO poultry	YES/NO poultry
red meat in small portions	NO red meat	NO red meat	NO red meat
grains, nuts, seeds	grains, nuts, seeds	grains, nuts, seeds	NO corn, oats and rye

**Table 2:** Diet types to manage RA and their effects on RA symptoms.

Diets	Effects on RA	References
Mediterranean diet	Physical function, Quality of life ↑ Inflammation ↓	[65]
	Physical function, Vitality ↑ Pain, stiffness, inflammation ↓	[59]
	Pain, inflammation ↓	[60]
Vegan diet	Pain, inflammation ↓	[60]
Vegeterian diet	Useful for the treatment of RA, weight loss ↓ Inflammation ↓	[59]
	Pain, inflammation ↓	[60,67]
Calorie restriciton/Fasting	Pain, stiffness, the number of tender and swollen joints ↓	[59]
	Pain, inflammation ↓	[60,68]
	Inflammation, obesity ↓	[67]
	Inflammation ↓	[69]
Elimination diet	Pain, inflammation ↓	[60,70]
	Inflammation ↓	[71]
	Inflammation, the number of tender ↓	[72]
